# Idiopathic Membranous Glomerulonephritis Leading to Secondary Polycythemia: A Rare Association

**DOI:** 10.7759/cureus.33985

**Published:** 2023-01-20

**Authors:** Prabhakar Yadav, Saurabh Pathak, Makkapalem s Malik

**Affiliations:** 1 Nephrology, Tata Main Hospital, Jamshedpur, IND

**Keywords:** polycythemia, receptor mutations, erythropoietin, membranous glomerulonephritis, nephrotic syndrome

## Abstract

Primary polycythemia is caused due to mutations in erythropoietin (EPO) receptor or Janus Kinase 2 (JAK2). Secondary polycythemia is seldom associated with renal diseases, such as adult polycystic kidney disease, kidney tumors (like renal cell carcinoma and reninoma), renal artery stenosis, and kidney transplant due to increased EPO production. Polycythemia associated with nephrotic syndrome (NS) is very rare. Here, we report a case with membranous nephropathy, the patient had polycythemia at presentation. Nephrotic range proteinuria causes nephrosarca leading to renal hypoxia which causes increased EPO and IL-8 production, this is proposed to cause secondary polycythemia in NS. The reduction of polycythemia following remission in proteinuria further suggests the correlation. The exact mechanism remains to be found out.

## Introduction

Polycythemia is defined as hemoglobin (Hb) of more than 16.5 g/dL in males and 16 g/dL in females or blood hematocrit of more than 49% in males and 48% in females (WHO 2016) [1]. Secondary polycythemia has been seen as a common association with renal conditions, which increases renal mass, such as adult polycystic kidney diseases, renal malignancies, and bilateral hydronephrosis. However, polycythemia in relation to nephrotic syndrome (NS) has rarely been described. Emanuel et al. in 1962 were the first one to describe this association [2]. We hereby report a case of secondary polycythemia from membranous nephropathy (MN), the etiopathogenesis of this phenomenon is not clear, hypovolemia, renal hypoxemia, and increased production of erythropoietin (EPO) by kidneys are most probable causes.

## Case presentation

A 61-year-old male presented with swelling of both lower limbs for one month, he is a known diabetic patient for the last 20 years and hypertensive for the last one year. He had no significant surgical history, he was a non-smoker and occasional drinker. Edema is of pitting type more in lower limbs and face, not associated with breathlessness, and has no postural association. He was on tab gliclazide 40 mg twice daily, tab metformin 500 mg thrice daily, tab losartan 50 mg twice daily, tab asprin 75 mg, tab atorvastatin 10 mg, metoprolol 25 mg twice daily, tab thyroxin 75 mcg once daily. He was on regular follow up and his diabetes and blood pressure were well controlled. There was no history of shortness of breath, chest heaviness, palpitations, skin eruptions, joint pain, hair fall, hemoptysis, and recurrent upper and lower respiratory tract symptoms. He had no history of reduced urine output, difficulty in micturition, or pyuria. His vital were as follows: pulse rate was 80 beats/min and blood pressure was 130/80 mmHg. On physical examination, he was tall and normal built, no pallor, no icterus, and edema present. His hemoglobin was 18.6 g/dL, hematocrit was 53.7%, urine routine microscopy suggestive of proteinuria without any active sediments, 24-hour urinary protein was 7.47 g/day, serum albumin was 2.52 g/dL, hyperlipidemia was present, and his Liver function tests were normal (Table 1).

**Table 1:** Lab reports on admission, three months, and six months. LDL: low-density lipoprotein; HbA1c: hemoglobin A1c; ANA: antinuclear antibody; ADNA: anti-double stranded deoxyribonucleic acid antibody; ANCA: antineutrophil cytoplasmic antibodies; RPR: rapid plasma reagin; HbsAg: hepatitis B surface antigen; HCV: hepatitis C virus; PSA: prostate-specific antigen; TSH: thyroid stimulating hormone

Parameters	At admission	3 months	6 months
Hemoglobin	18.6 g/dL	16.6 g/dL	13.6 g/dL
Red blood cell count	6.2 million/mm^3^	5.5 million/mm^3^	5.0 million/mm^3^
Hematocrit	53.7%	50.7%	48.7%
Total leucocyte count	15,700/mm^3^	12,700/mm^3^	10,500/mm^3^
Platelets count	298,000	250,000	200,000
Urine culture	Sterile	Sterile	Sterile
Urine protein creatinine ratio	7.478 g/day	2.5 g/day	500 mg/day
Creatinine	0.87 mg/dL	0.6 mg/dL	0.5 mg/dL
Serum bilirubin	0.7 mg/dL	-	-
Serum albumin	2.52 g/dL	3.25 g/dL	4.06 g/dL
LDL cholesterol	149.36 mg/dL	100 mg/dL	80 mg/dL
Triglycerides	307 mg/dL	230 mg/dL	140 mg/dL
Uric acid	6.8 mg/dL	-	-
Fasting blood sugar	140 mg/dL	130 mg/dL	114 mg/dL
Post-prandial blood sugar	204 mg/dL	180 mg/dL	140 mg/dL
HbA1c	8.9	7.0	6.5
Serum calcium	8.58 mg/dL	-	-
Serum erythropoietin	14 mU/mL	-	20 mU/mL
JAK2 V617F gene mutation	Not detected	-	-
ANA/ADNA	Negative	-	-
ANCA	Negative	-	-
RPR	Negative	-	-
HbsAg/HCV/ HIV	Negative	-	-
PSA	3.21	-	-
TSH	7.56	-	-
Bone marrow	Mildly hypercellular, normal erythroblast, mild lymphocytosis, megakaryocytes normal	-	-
USG whole abdomen	Normal size kidney, no hepatosplenomegaly	-	-

**Figure 1:**
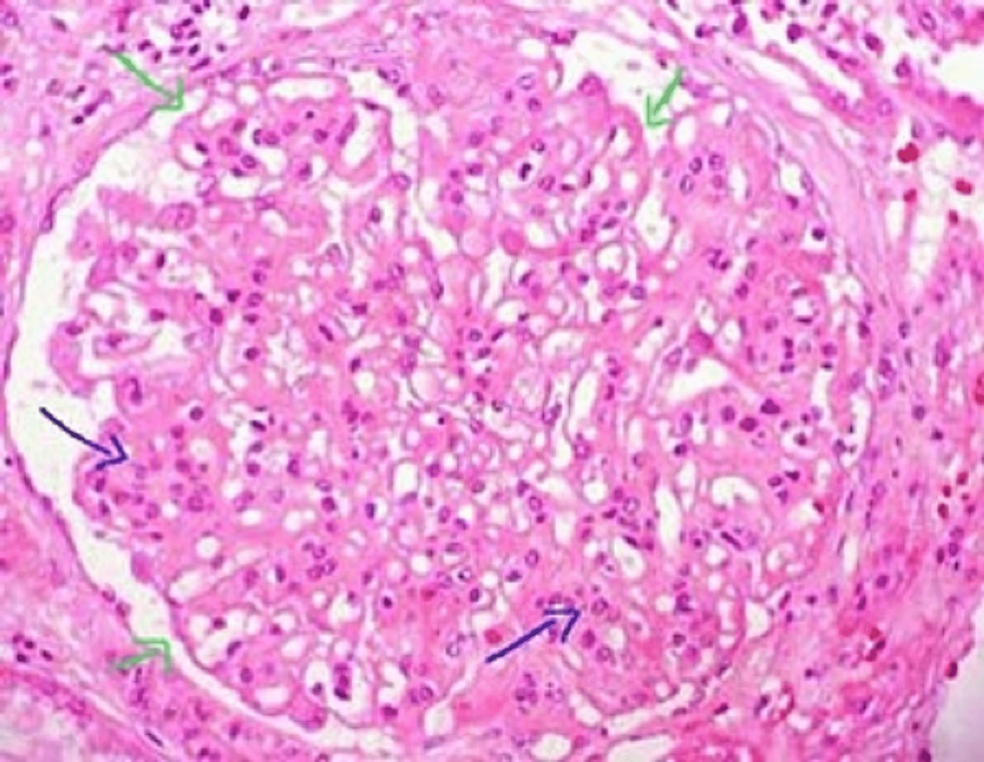
Histopathology report of renal biopsy (light microscopy). The image shows the following: (1) glomerulus - glomeruli appeared enlarged and diffuse thickening of capillaries which showed membrane texture alterations and intramembranous mottling discernible in methenamine stained sections, no evidence of nodule formation, intracapillary thrombi and crescent formation. (2) Tubules - tubular atrophy and interstitial fibrosis in 10-12% of sampled cortex. (3) Arteries - mild medial thickness and arterioles revealed focal hyalinosis lesions. (4) Immunofluorescence - IgG- 3+, IgA, IgM and C1q Negative, Kappa and lambda- 3+, staining for PLA2R, THSD7A, NELL-1, EXT-1 and Sema 3b-negative. Here, the green arrow shows thickened capillary wall and the blue arrow shows mesangial hypercellularity.

A provisional diagnosis of polycythemia with nephrotic syndrome was made and was investigated further. Evaluation for secondary causes of membranous nephropathy was done his antinuclear antibody (ANA), rapid plasma reagin (RPR)/syphilis antibodies, hepatitis C virus (HCV), hepatitis B surface antigen (HbsAg), and HIV tests were negative. His anti-neutrophil cytoplasm antibody (ANCA) and anti-glomerular basement membrane (anti-GBM Ab) were negative and his C3 and C4 levels were normal. CT abdomen and chest were negative for any overt malignancies or hepatosplenomegaly. The patient’s serum EPO level was 14.00 IU/L (normal: 5-30 IU/L) with respective hemoglobin of 18.6 g/dL, JAK2 exon12 and V617F mutations for polycythemia vera (PV) were negative. Hematology evaluation concluded that primary polycythemia is unlikely. Bone marrow was suggestive of normal erythropoiesis with mild erythroid hyperplasia, 2-D echocardiography showed no regional wall motion abnormality with ejection fraction of 55%. The ultrasonography showed bilateral normal-sized kidneys and no hepatosplenomegaly. Renal biopsy was planned in view of nephrotic range proteinuria. Renal biopsy findings are shown in Figure 1.

A kidney biopsy revealed membranous nephropathy. Staining for PLA2R and THSD7A, NELL-1, EXT-1, and Sema 3b were negative within the glomerular deposits. He was put on a modified Ponticelli regimen for six months and gradually his proteinuria and hemoglobin settle down.

## Discussion

Nephrotic syndrome with polycythemia is a rare condition and it is rarely reported. Most cases of NS and polycythemia reported correspond to focal segmental glomerulosclerosis (FSGS), mainly associated with polycythemia vera (PV) [3-5], one isolated case of FSGS with secondary polycythemia had also been reported by Yin et al. in 2014 [6]. Sharma et al. also reported a similar case of FSGS in association with PV, as reported by him, the hemodynamic variations in the PV as well as renal vasodilation and increased renal blood flow stimulate renal glomerulosclerosis [7]. Ferrario et al. suggested that the probable etiology of FSGS is hemodynamic-related glomerular injury causing mesangial cell activation, which in turn causes increased extracellular matrix production [8]. Balal et al. and Heras et al. also reported two cases of minimal change disease (MCD) and polycythemia in 2004 and 2016, respectively [9,10]. Secondary polycythemia is seldom associated with renal disorders which increase EPO production such as kidney tumors, polycystic diseases, bilateral hydronephrosis, post-kidney transplantation, renal artery stenosis, and Bartter syndrome, and it is seldom reported with nephritic syndrome, nephrosclerosis, chronic glomerulonephritis and MN [11]. Stack et al. were the first to report a case of erythrocytosis associated with idiopathic membranous glomerulonephritis (IMN) [12]. Chen et al. in 1990 also reported a case of secondary polycythemia associated with MN in a 61-year-old male [13]. Khan et al. reported a similar case of secondary polycythemia associated with IMN in a 44-year-old male in 1993 [14]. Lim et al. in 2000 also reported a similar case of secondary polycythemia associated with IMN in a 58-year-old male [15]. PV was excluded in our patient as serum erythropoietin was normal and JAK2 mutation was negative and bone marrow test was suggestive of mild erythroid hyperplasia so secondary polycythemia was considered. There was no evidence of secondary causes of MN in blood investigations and on tissue staining in renal biopsy, therefore, a diagnosis of secondary polycythemia due to IMN was made.

Polycythemia is seldom seen in patients with MN, renal hypoxemia due to severe nephrosis leading to increased EPO production is the main stimulus. The etiology for polycythemia in chronic kidney diseases proposed were as follows: increased EPO production due to renal hypoxia, increased sensitivity to EPO, and abnormal feedback regulation of erythrocytosis [16-19]. The increased activity of renin-angiotensin system was involved in this process, and the beneficial effect of angiotensin-converting enzyme (ACE) inhibitors or angiotensin II type I receptor blockers in control of polycythemia after renal transplantation was the basis for this [18,19]. However, the exact stimuli leading to polycythemia in the presence of renal abnormalities are still not clear. Polycythemia makes the blood hyperviscous and increases the risk of stroke and thromboembolism in such patients due to hypercoagulability in setting of severe hypoalbuminemia. Hence, prophylactic anti-coagulation should strongly be considered in these patients till remission of proteinuria and erythrocytosis. The result of polycythemia was different in various case reports reported, in some patients, the polycythemia remitted with a reduction in glomerular filtration rate which results in reduced proteinuria and thus reduced EPO production [14,16], but in others, the polycythemia remained as such despite renal failure and reduced proteinuria [2,13], phlebotomy was done in such patients and were put-on long-term anti-coagulants for reduction of risk of hyperviscosity and thromboembolism [13,14]. In our patient, phlebotomy was performed, and an anti-coagulant was added along with cyclophosphamide and prednisolone. The hematocrit and red cell count gradually declined with reduction of proteinuria. The serum erythropoietin was normal at the time of diagnosis, and it remains normal throughout the course of treatment and at resolution of polycythemia. The arterial oxygen saturation was within normal range at the time of polycythemia, which could be due to renal hypoxia only. Renal perfusion defect because of nephrosarca causes increased erythropoietin level, with resolution of proteinuria, the tissue edema resolved and the stimulation for erythropoietin production and sensitivity to erythropoietin decreases. The effect of ACE inhibitors on the renin-angiotensin complex or erythropoietin and the cytotoxic effect of cyclophosphamide on bone marrow hematopoietic stem cells cause further resolution of polycythemia. These factors have add-on effect on the remission of polycythemia and proteinuria.

## Conclusions

Polycythemia is a rare complication due to NS. FSGS was the most common form of NS reported in association with PV. FSGS, MCD, and a few cases of MN had also been reported in association with secondary polycythemia. We reported here a case of polycythemia due to IMN, a rare association. Early diagnosis and prompt management with cyclophosphamide, prednisolone, angiotensin receptor blocker, and anticoagulants resolve proteinuria and polycythemia, and hence risk of thromboembolism is reduced.

## References

[1] Barbui T, Thiele J, Gisslinger H (2018). The 2016 WHO classification and diagnostic criteria for myeloproliferative neoplasms: document summary and in-depth discussion. Blood Cancer J.

[2] EM DA, WE FJ (1962). Erythrocytosis associated with the nephrotic syndrome. JAMA.

[3] Okuyama S, Hamai K, Fujishima M, Ohtani H, Komatsuda A, Sawada K, Wakui H (2007). Focal segmental glomerulosclerosis associated with polycythemia vera: report of a case and review of the literature. Clin Nephrol.

[4] Martín JS, Suárez LG, Martín FG (2010). Focal and segmental glomerulosclerosis associated with polycythemia vera. [Article in Spanish]. Nefrologia.

[5] Ulusoy S, Ozkan G, Sönmez M, Mungan S, Kaynar K, Cansiz M, Kazaz N (2010). Absence of hypoalbuminemia despite nephrotic proteinuria in focal segmental glomerulosclerosis secondary to polycythemia vera. Intern Med.

[6] Yin Q, Yang Y, He T (2014). A case of focal segmental glomerulosclerosis syndrome secondary to high-altitude polycythemia. Ren Fail.

[7] Sharma RK, Kohli HS, Arora P (1995). Focal segmental glomerulosclerosis in a patient with polycythemia rubra vera. Nephron.

[8] Ferrario F, Rastaldi MP, Pasi A (1999). Secondary focal and segmental glomerulosclerosis. Nephrol Dial Transplant.

[9] Balal M, Seyrek N, Karayaylali I, Paydas S (2004). A unique form of polycythemia associated with minimal change disease. Med Princ Pract.

[10] Heras M, Saiz A, Rosado B (2016). Association of minimal-change disease and polycythemia in a very elderly patient. Nefrologia.

[11] Karunarathne S, Udayakumara Y, Govindapala D, Fernando H (2012). Medullary nephrocalcinosis, distal renal tubular acidosis and polycythaemia in a patient with nephrotic syndrome. BMC Nephrol.

[12] Stack JI, Zabetakis PM (1979). Erythrocytes associated with idiopathic membranous glomerulopathy. Clin Nephrol.

[13] Chen YC, Yeh JC, Chen HS, Hsu HC (1990). Secondary polycythemia associated with membranous nephropathy. Clin Nephrol.

[14] Khan IH, Simpson JG, MacLeod AM, Catto GR (1993). Secondary polycythaemia associated with idiopathic membranous nephropathy. Nephron.

[15] Lim CS, Jung KH, Kim YS, Ahn C, Han JS, Kim S, Lee JS (2000). Secondary polycythemia associated with idiopathic membranous nephropathy. Am J Nephrol.

[16] Basu TK, Stein RM (1974). Erythrocytosis associated with chronic renal disease. Arch Intern Med.

[17] Sonneborn R, Perez GO, Epstein M, Martelo O, Pardo V (1977). Erythrocytosis associated with the nephrotic syndrome. Arch Intern Med.

[18] Danovitch GM, Jamgotchian NJ, Eggena PH, Paul W, Barrett JD, Wilkinson A, Lee DB (1995). Angiotensin-converting enzyme inhibition in the treatment of renal transplant erythrocytosis. Clinical experience and observation of mechanism. Transplantation.

[19] Navarro JF, García J, Macía M (1998). Effects of losartan on the treatment of posttransplant erythrocytosis. Clin Nephrol.

